# Probing Protein Sequences as Sources for Encrypted Antimicrobial Peptides

**DOI:** 10.1371/journal.pone.0045848

**Published:** 2012-09-28

**Authors:** Guilherme D. Brand, Mariana T. Q. Magalhães, Maria L. P. Tinoco, Francisco J. L. Aragão, Jacques Nicoli, Sharon M. Kelly, Alan Cooper, Carlos Bloch

**Affiliations:** 1 Laboratório de Espectrometria de Massa, Embrapa Recursos Genéticos e Biotecnologia, Brasília, Distrito Federal, Brazil; 2 Laboratório de Transferência e Expressão de Genes, Embrapa Recursos Genéticos e Biotecnologia, Brasília, Distrito Federal, Brazil; 3 Departamento de Microbiologia, Instituto de Ciências Biológicas, Universidade Federal de Minas Gerais (UFMG), Belo Horizonte, Brazil; 4 Institute of Molecular, Cell & Systems Biology, University of Glasgow, Glasgow, Scotland, United Kingdom; 5 School of Chemistry, University of Glasgow, Glasgow, Scotland, United Kingdom; Russian Academy of Sciences, Institute for Biological Instrumentation, Russian Federation

## Abstract

Starting from the premise that a wealth of potentially biologically active peptides may lurk within proteins, we describe here a methodology to identify putative antimicrobial peptides encrypted in protein sequences. Candidate peptides were identified using a new screening procedure based on physicochemical criteria to reveal matching peptides within protein databases. Fifteen such peptides, along with a range of natural antimicrobial peptides, were examined using DSC and CD to characterize their interaction with phospholipid membranes. Principal component analysis of DSC data shows that the investigated peptides group according to their effects on the main phase transition of phospholipid vesicles, and that these effects correlate both to antimicrobial activity and to the changes in peptide secondary structure. Consequently, we have been able to identify novel antimicrobial peptides from larger proteins not hitherto associated with such activity, mimicking endogenous and/or exogenous microorganism enzymatic processing of parent proteins to smaller bioactive molecules. A biotechnological application for this methodology is explored. Soybean (*Glycine max*) plants, transformed to include a putative antimicrobial protein fragment encoded in its own genome were tested for tolerance against *Phakopsora pachyrhizi*, the causative agent of the Asian soybean rust. This procedure may represent an inventive alternative to the transgenic technology, since the genetic material to be used belongs to the host organism and not to exogenous sources.

## Introduction

Biologically active peptides encrypted in either precursor or mature proteins can be unveiled by endogenous enzymatic hydrolysis or proteolytic cleavage during digestive processes [1], [2], [3], [4]. A number of specific examples describing the enzymatic release of bioactive peptides from longer polypeptide chains have been extensively documented [5], [6], [7], [8]. Classic examples demonstrate the breadth and the physiological relevance of these metabolic processes. These include the cleavage of high molecular weight kininogen by blood plasma kallikrein in mammals, leading to the generation of the vasoactive bradykinin [9]; frog skin antimicrobial peptides synthesized as highly conserved preproprotein, yielding mature molecules after a typical prohormone signal processing [10]; and hemorphins, the earliest opioid-like peptides obtained from proteolytic degradation of haemoglobin [8].

In a biotechnological context, fermentation of milk with proteolytic starter cultures or proteolysis by enzymes derived from microorganisms, plants and animal digestion have proven to be efficient approaches to obtain an array of bioactive peptides. These strategies have been applied successfully since the early days of the dairy industry, exploiting the same principles as the precursor molecule processing but with exogenous enzymatic sources.

Considering the mass of genomic data now available, we envisioned the possibility of exploring theoretical protein fragmentation in search of novel biologically active peptides encrypted in expressed proteins, regardless of their structure and function in the host organisms. The primary aim is to emulate the exogenous proteolytic features and biotechnological principles common to the dairy and other microorganism-based processing industries to predict, synthesize and test new molecules that might otherwise be latent within the three-dimensional folds of numerous proteins. The present manuscript introduces a methodology based on the *in silico* filtering of putative antimicrobial fragments of proteins in association with biological tests that allows us to categorize groups of peptides with different degrees of affinity to biological membranes and to select novel antimicrobial peptide sequences encoded within much larger proteins.

We have chosen microbicidal activity as the proof-of-principle of this concept, since naturally occurring antimicrobial peptides represent an ancient and pervasive part of the innate immune system of organisms from different kingdoms, constituting the first barrier against the invasion of pathogens [11]. Various studies have demonstrated that these peptides usually induce disturbances in biological membranes ultimately leading to cell death [12], [13]. Despite considerable efforts, we still lack a comprehensive understanding of the major variables that underlie the interactions of peptides with biological membranes and/or a general framework to evaluate functional similarities among peptides.

We report here the experimental basis for the prediction and evaluation of antimicrobial peptides derived from various protein sequences from different organisms. An exploratory software procedure, named Kamal, was developed in-house as a primary search tool to uncover putative antimicrobial sequences from proteins based on physicochemical similarity to a sample of known antimicrobial peptides. Fifteen protein fragments and eleven naturally occurring peptides were chemically synthesized and tested for antimicrobial activities. Biophysical assays were conducted to gain a deeper understanding of the peptide/membrane interactions. Peptide interactions with large unilamellar vesicles (LUVs) composed of DMPC and 2∶1 DMPC:DMPG were systematically investigated using differential scanning calorimetry (DSC), to probe effects on the thermotropic phase behaviour of membranes, and circular dichroism (CD) to determine the effects of membrane interaction on peptide secondary structure. A principal component analysis (PCA) was applied to the resulting data. The effects produced by these peptides on the main phase transition of model membranes were correlated to the degree of peptide α-helical contents attained upon titration with LUVs of the same compositions, as evaluated by CD. The results on model membranes were also correlated to antimicrobial potencies against the pathogenic bacteria *Escherichia coli*, *Staphylococcus aureus* and *Pseudomonas aeruginosa* and the phytopathogenic bacterium *Xanthomonas axonopodis* pv. *glycines*. Selected peptides, including fragments of soybean proteins, were tested for inhibitory effects on the germination of *Phakopsora pachyrhizi* spores on the leaf surface of the soybean plant (*Glycine max*). Additionally, *G. max* plants transformed with a fragment of the enzyme D-myo-inositol 3-phosphate synthase were artificially inoculated with *P. pachyrhizi* spores showing evident tolerance to the fixation of Asian rust spores. The molecules resulting from this procedure were named intragenic antimicrobial peptides (abbreviated IAPs).

## Results

### 1. Selection of Intragenic Antimicrobial Peptides (IAPs)

Candidate IAPs were selected by scanning publicly available protein sequences using a bioinformatic tool (Kamal). Details of the software and its implementation will be presented elsewhere but, briefly, Kamal performs an *in silico* digestion of proteins over a sliding window into peptide fragments of definable length. These are then filtered using specific sequence-based criteria in order to identify a set of peptide candidates with potentially desirable activities. This is illustrated in Table 1 by application to sequences from the soybean (*Glycine max*) genome project [14]. Here, Table 1, we compare calculated physicochemical properties of protein fragments to a reference range of values extracted from a sample of antimicrobial peptides (AMPs) of the Antimicrobial Peptide Database, APD [[15]; (http://aps.unmc.edu/AP/main.php)]. The list of reference AMPs and their calculated physicochemical properties are available as Table S1. Putative IAPs from the fore mentioned database were selected if all evaluated physicochemical properties of each protein fragment were within the reference peptide set limits, otherwise were discarded. Reference values chosen here relate to a simple set of physicochemical properties of relevance to peptide interactions with membranes as putative descriptors of peptide activity [12], [16], [17], [18]. Approximately 500 putative IAPs were filtered from the *G. Max* expressed sequence tags (EST) database (Table S1). In addition, for comparison, five hundred additional peptides from 20 to 30 amino acids were selected at random from proteins of the same database (Table S1).

**Table 1:** Peptide search criteria and illustrative data for the filtering of putative IAPs from the soybean (*Glycine max*) genome project [14].

Kamal search criteria	Kamal Filtering Range	AMPs from the APD	Putative IAPs from *G.max*	Random fragments from *G.max*	PCA loadings		
					PC1	PC2	PC3
Net charge (pH 7.0)	+1 to +6	2.32 (1.73)	1.70 (0.95)	−0.13 (3.17)	−0.153	−0.518	0.277
Monoisotopic mol. mass	2200 to 3200 Da	2413.95 (782.17)	2672.86 (266.15)	2759.38 (249.66)	–	–	–
Isoelectric point (Bjellqvist [51])	8 to 11	9.01 (1.33)	9.13 (0.85)	6.65 (2.57)	−0.211	−0.513	0.261
Hydrophobicity (TM scale [49])	−1 to +2	0.00 (0.40)	−0.13 (0.19)	−0.47 (0.36)	−0.458	0.143	−0.103
Hydrophobic moment (TM scale [49])	0.3 to 1.4	0.66 (0.26)	0.50 (0.12)	0.35 (0.17)	−0.296		−0.177
Min hydropathy (K-D Hydropathy plot [50])	−1.5 to 1	−0.22 (0.84)	−0.79 (0.44)	−1.29 (0.77)	−0.431	0.147	−0.140
Max hydropathy (K-D Hydropathy plot [50])	+0.5 to +3	1.64 (0.61)	1.36 (0.58)	0.90 (0.68)	−0.371		−0.313
Aggregation (Nav4SS Aggrescan [16])	0 to +40	20.71 (21.69)	16.42 (9.61)	−7.15 (22.77)	−0.469		
GORIV Alpha helix [48]	-	18.92 (20.69)	18.78 (19.49)	12.58 (16.72)		−0.481	−0.532
GORIV Extended strand [48]	-	47.64 (14.06)	39.87 (11.22)	59.37 (15.04)	0.182	0.395	
GORIV Random coil [48]	-	33.44 (17.05)	41.35 (19.04)	28.05 (14.16)	−0.233	0.171	0.638

-Parameter not used.

* no Cys or Pro.

**Figure 1:**
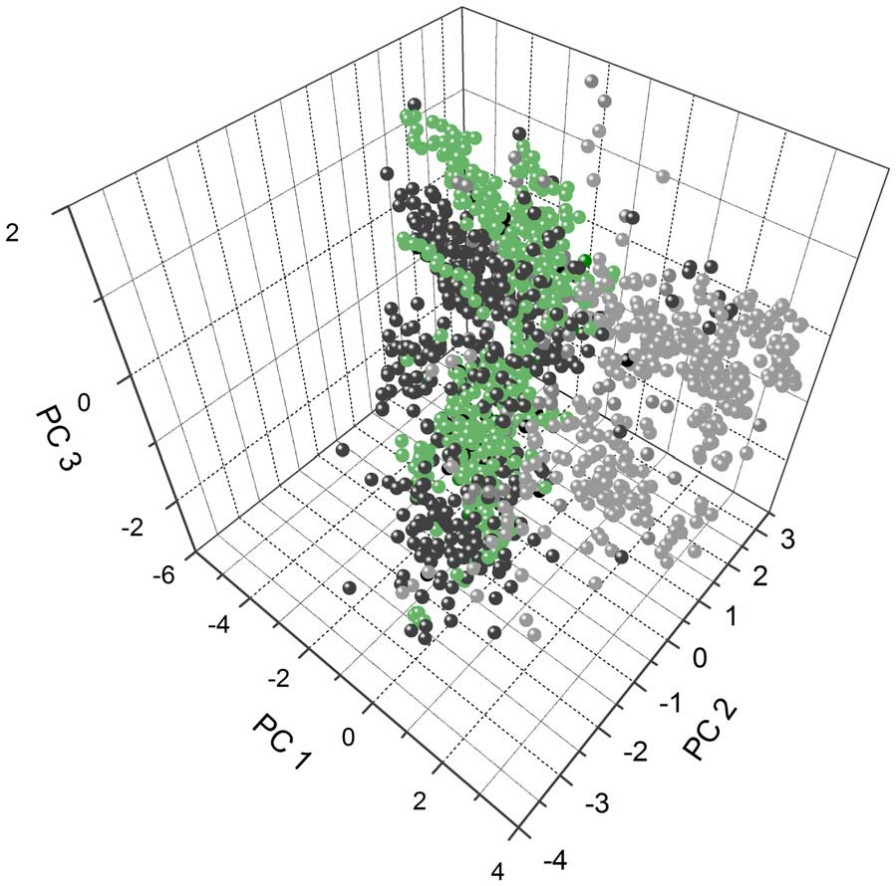
Peptides filtered by Kamal are physicochemically more similar to a sample of antimicrobial peptides than randomly chosen molecules from the same EST database. A principal component analysis was applied to calculated physicochemical properties (see Table S1) of five hundred randomly chosen protein fragments and five hundred putative IAPs filtered from an EST database of *G. max* proteins [14], represented as green and grey spheres respectively, as well as naturally occurring antimicrobial molecules from the Antimicrobial Peptide Database [15], in black. Component loadings are available in Table 1.

From the summary data (Table 1) it is clear that the average estimated parameters for the filtered *G. Max* IAP candidates are generally closer to the APD than to the random set of peptides, as is inevitable from the selection criteria used by Kamal. However, simple averages just paint a crude picture. A principal component analysis applied to the resulting data (Fig. 1) demonstrates more clearly how the putative IAPs filtered by Kamal (green spheres) show superior overall physicochemical similarity to the sample of AMPs (black spheres) than randomly chosen fragments of soy proteins (grey spheres). Thus, if the premises are correct, these have a higher probability of being antimicrobial. The first principal component results mainly from various hydrophobicity-derived physicochemical properties, while the second and third by peptide charge and secondary structure parameters (Table 1). Although the coincidence of the AMP and IAP clusters (Fig. 1) is far from exact, this putative IAP set should contain promising antimicrobial candidates.

To verify the actual antimicrobial potential of the sequences filtered by Kamal, fifteen putative IAPs were arbitrarily selected from a more extensive search covering a range of organisms (Table 2), and chemically synthesized. Additionally, eleven AMPs from frog skin secretions described by our group were used as controls for naturally occurring molecules, together with the cell-penetrating peptide penetratin, which is also antimicrobial [19]. Table 2 demonstrates that six out of the fifteen synthesized putative IAPs did present a minimal inhibitory concentration (MIC) against at least one of the tested microorganisms, the human pathogens *E. coli*, *S. aureus* and *P. aeruginosa* and the phytopathogenic bacterium *X. axonopodis* pv. *glycines*. Two peptides, Q6TV81(25–52) from the ORF 107 of the bovine papular stomatitis virus and A5LDU0(184–211), a fragment of the enzyme pseudouridine synthase of *Streptococcus pneumoniae* SP3-BS71, had MICs comparable to naturally occurring antimicrobial peptides. Building on this, we have utilised biophysical assays on peptide and membrane interactions in order to identify similarly acting molecules and provide insights on the physicochemical requirements for peptide function.

**Table 2:** Peptides primary structures, source proteins, organisms and minimum inhibitory activity (MIC) against pathogenic bacteria.

Peptide name*	Primary structures		MICs (µM)				
		Source protein	Source organism	*Xanthomonas axonopodis pv. glycines*	*Escherichia* *coli*	*Staphylococcus aureus*	*Pseudomonas aeruginosa*
P61458(35–60)	FKQFHFKDFNRAFGFMTRVALQAEKL	Pterin-4-alpha-carbinolamine	*Mus musculus*	40	40	NDA	80
B0CZJ3(104–130)	IAAAQRITSGAADIAINWAGGLHHAKK	Histone deacetylase complex	*Laccaria bicolor*	NDA	NDA	NDA	NDA
A4HW34(187–217)	LVQRFHAYLHKFREAFMNVGAAAAVEGTKAA	Glutathione synthetase	*Leishmania infantum*	37	NDA	NDA	NDA
Q8RW88(70–95)	GHRGALKDWVQAAGGAVAAFDFTTKG	Alpha-amylase	*Citrus reticulata*	NDA	NDA	NDA	NDA
Q6TV81(25–52)	AAAAAAAIKMLMDLVNERIMALNKKAKK	ORF107 virion morphogenesis	*Bovine papular stomatitis virus*	<1	10	20	20
O43312(33–62)	FINKAGKLQSQLRTTVVAAAAFLDAFQKVA	Metastasis suppressor protein 1	*Homo sapiens*	80	40	NDA	NDA
Q8KG25(327–351)	FVTNSKRLAEGIEKGVGNSILIKVN	Enolase 2	*Chlorobium tepidum*	NDA	NDA	NDA	NDA
P94692(929–955)	KLKKLLAGQKDGLLGQIAAMSDLYTKK	Pyruvate-ferredoxin oxidoreductase	*Desulfovibrio africanus*	NDA	NDA	NDA	NDA
B4FGE3(22–37)	KAGLQFPVGRIARFLK	Histone H2A	*Zea mays*	NDA	NDA	NDA	NDA
A3KLW0(117–136)	FKALRALRLEDLRIPTSYIK	Rubisco large chain	*Anisophyllea pomifera*	NDA	NDA	NDA	NDA
Q7YRI0(9–28)	LAKRRVLTLLRQLRRVSPSS	Interferon alpha	*Bos taurus*	<2	14	NDA	NDA
gb|ACU24018.1|(73–101)	GLWQIFSSKEEGKDNSQQKSKGDQAKEL	Unknown protein	*Glycine max*	NDA	NDA	NDA	NDA
gb|AAD22970.1|(120–148)	VWTTAMEKSSAANFSMSRNQRRSSLHSL	Trehalase 1 GMTRE1	*Glycine max*	NDA	NDA	NDA	NDA
Q9XEY7(120–148)	SLWKNLSRKISGAVKAQPDLHTLLPLPGS	Trehalase 1 GMTRE1	*Glycine max*	NDA	NDA	NDA	NDA
A5LDU0(184–211)	GKFHQIKKMFLSVGVKVTSLKRIQFGDF	Pseudouridine synthase	*Streptococcus pneumoniae*	10	20	NDA	20
DS 01	GLWSTIKQKGKEAAIAAAKAAGQAALGAL	None	*Phyllomedusa oreades*	<1	1	11	6
Nattererin-1	GLKDMIKNLAKEAAVKLAGAVINKFSPQPQ	None	*Eupemphix nattereri*	<1	5	40	10
PS -2	FLSLIPHAINAVSTLVHHF	None	*Phyllomedusa hypochondrialis*	121	15	NDA	NDA
DS 01 (1–12)	GLWSTIKQKGKE**	DS 01	*Phyllomedusa oreades*	NDA	NDA	NDA	NDA
Syphaxin	GVLDILKGAAKDLAGHVATKVINKI	None	*Leptodactylus syphax*	25	12	50	100
HSP-4	GIGDILKNLAKAAGKAALHAVGESL	None	*Hypsiboas punctatus*	<2	19	19	77
Pseudin B	GLNTLKKVIQGLHEVIKLVNNHA	None	*Pseudis bolbodactyla*	<2	6	6	50
Phes	FFFDTLKNLAGKVIGALT	None	*Hypsiboas punctatus*	33	62	62	125
Magainin-2 amide	GIGKFLHSAKKFGKAFVGEIMNS	None	*Xenopus laevis*	13	13	NDA	26
Hyposin HA-6	LRPAILVRVKGKGL	None	*Phyllomedusa hypochondrialis*	42	NDA	NDA	NDA
Penetratin	RQIKIWFQNRRMKWKK	Antennapedia complex	*Drosophila melanogaster*	<2	7	28	2

NDA  =  Non-detectable activity.

* publication names, UniprotKB or genebank entries followed by the first and last residues in brackets.

** free carboxy-terminus peptide.

### 2. Categorization of Peptide Interaction with Model Phospholipid Vesicles by DSC

A number of studies elsewhere show that membrane active compounds affect the thermotropic behaviour of phospholipid vesicles in ways that can be related to their mechanism of action [20], [21]. Consequently we here used DSC to study the effect of IAPs and naturally occurring antimicrobial peptides on the main phase transition of LUVs composed of DMPC and 2∶1 DMPC:DMPG.

DSC heating scans of DMPC LUVs showed the anticipated endothermic transitions typical of the well-characterized gel to liquid crystalline (P’_β_→Lα) thermal phase transitions [21], [22]. Thermograms were deconvoluted and fit to a non-two state model revealing two peaks, a broad (Tm = 23.4°C) and a sharp component (Tm = 24.1°C) in agreement with earlier work [22]. The incorporation of DMPG to the mixture, which by itself has a transition temperature of 23.9°C, resulted in transition temperatures of 23.6°C for the broad component and 24.3°C for the sharp component as well as comparable transition enthalpies (Table S2) [23].

**Figure 2:**
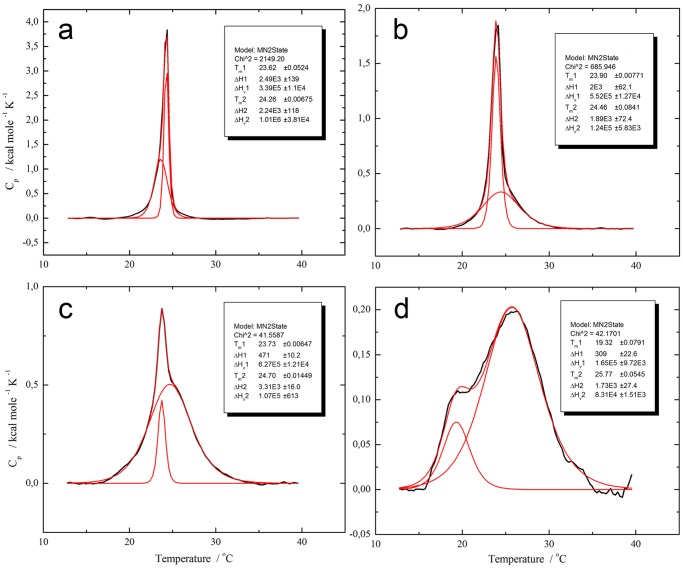
Thermal scans of 2∶1 DMPC:DMPG LUVs enriched with increasing concentrations of DS 01 show the effect of frog skin antimicrobial peptides on the main phase transition of phospholipids. Non two-state model fitting of the P’_β_→Lα phase transition of a solution of 0.5 mM 2∶1 DMPC:DMPG LUVs enriched with a) pure phospholipids, b) 1 mol% DS 01, c) 2 mol% DS 01 and d) 4 mol% DS 01.

The effect of the frog skin antimicrobial peptides on the thermotropic phase behavior of phospholipid LUVs has been well described [20] and is illustrated here (Fig. 2) by the case of AMP DS 01. (See Fig. S1 for details). The effects of additional twenty-five peptides at 4 mol% on the main phase transition of LUVs were studied and endotherms were also fit to broad and sharp components (Table S2). As might be expected, peptides varied in their ability to promote changes on the main phase transition of LUVs, as shown for example in Fig. S2 for the frog skin peptide PS-2, and the IAPs Q6TV81(25–52), Q8KG25(327–351) and A5LDU0(184–211).

**Figure 3:**
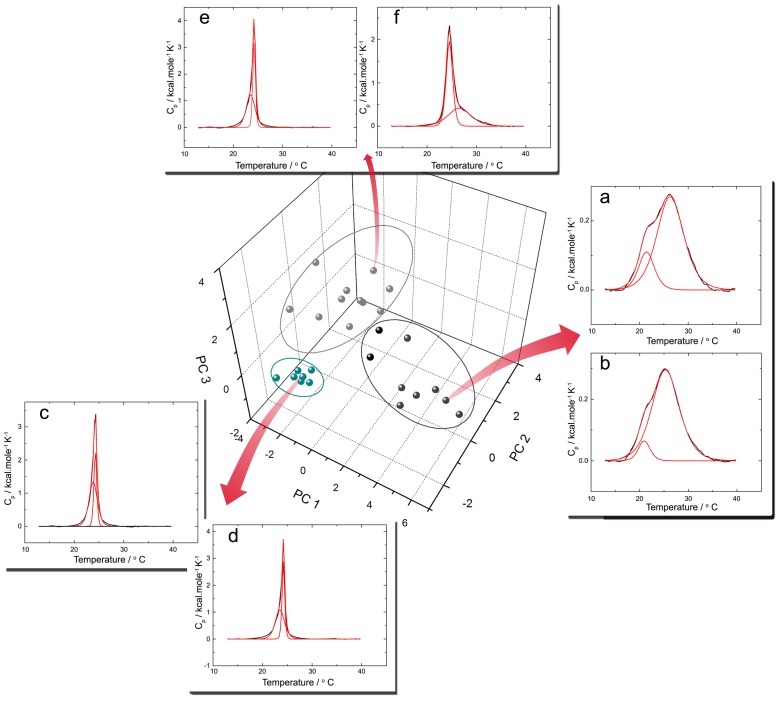
Principal component analysis of the model fitted main phase transitions of LUVs added with peptides at 4 mol% highlight similarities on membranes thermal behaviors. A matrix describing the effect of peptides on the transition temperature (Tm), enthalpy (ΔH) and cooperativity (ΔH_VH_) of the broad and sharp components of DMPC and 2∶1 DMPC:DMPG LUV thermal transitions was assembled and standardized (Table S2) and a PCA was applied to the resulting data. Additionally, the coordinates of peptides in the first three principal components were submitted to a mixture modeling clustering algorithm, resulting in an optimal of three peptide clusters, shown here in different colors (Fig. S3). Thermograms demonstrating the effect of selected peptides on the main phase transition of membranes are shown as representative examples: HSP-4 on (a) 2∶1 DMPC:DMPG and (b) DMPC LUVs represents cluster 3, in black, the IAP Q8RW88(70–95) on (c) 2∶1 DMPC:DMPG and (d) DMPC LUVs represents cluster 1, in green, and penetratin added to (e) DMPC and (f) 2∶1 DMPC:DMPG LUVs represents cluster 2, in grey. Group borders are illustrational.

Although visual inspection allows the identification of similar peptide-induced membrane phase transitions, with such a wealth of data it can be difficult to find meaningful correlations in a non-subjective manner. Principal component analysis (PCA) is a valuable descriptive tool for dimensionality reduction that does not require continuous or normally distributed variables [24]. The application of PCA resulted in three principal components that can jointly explain approximately 80% of the data variance. This is illustrated graphically in Fig. 3, with full numerical details of principal component loadings given in Table S2. Peptides that have a positive projection on the first principal component, such as DS 01 for example, induced the most significant changes on the main phase transition of vesicles of both compositions. Thermograms involving peptide HSP-4 are used as representative examples in Fig. 3. Principal component 2 is dominated by variables extracted for the interaction of peptides with 2∶1 DMPC:DMPG LUVs (Table S2). Therefore, molecules that affected the main phase transition of negatively charged but not neutral membranes are distributed on it. Fig. 3 insets e and f illustrate the example of penetratin, which did not induce changes in the thermal profile of DMPC LUVs, but modified the sharp and broad components of 2∶1 DMPC:DMPG LUVs. Principal component 3 has its highest loadings attributed to variables such as the enthalpy and van’t Hoff enthalpy associated with the broad component for 2∶1 DMPC:DMPG LUVs, and the peptide Phes has the highest projection on it (Table S2).

As illustrated in Fig. S3, and indicated in Fig. 3, peptides in the space of the first three principal components were best clustered in three groups [25]. Cluster 1, colored in green in Fig. 3, is formed by peptides that did not induce noticeable changes in the P’_β_→Lα phase transition for either membrane compositions. Cluster 2, colored in gray, is populated by peptides that affected the phase transitions of 2∶1 DMPC:DMPG LUVs but presented little or no effect on DMPC vesicles. Peptides colored in black induced profound changes in the thermotropic behavior of vesicles of both compositions. A detailed list of peptide cluster memberships can be found on Table S4.

### 3. Peptide Secondary Structure

Far UV CD spectra were acquired for peptides at 20 µM in buffer and in the presence of DMPC or 2∶1 DMPC:DMPG LUVs at 1 mol% peptide/phospholipids [26]. Peptides presented different degrees of secondary structures upon LUVs addition. CD spectra in buffer were typical of unordered structures for most samples, with the exception of the IAP Q6TV81(25–52) (Table S3). Following the addition of LUVs, some peptides changed conformation resulting in spectral features characteristic of α-helical structures [27]. Data for each CD spectrum was used for the estimation of the percentage of an α-helix, as shown in Table S3 [28], [29]. Peptides A5LDU0(184–211) and gb|AAD22970.1|(120–148) exhibited far UV CD spectra consistent with the spectral characteristics of β-sheet structures and these were considered separately.

**Figure 4:**
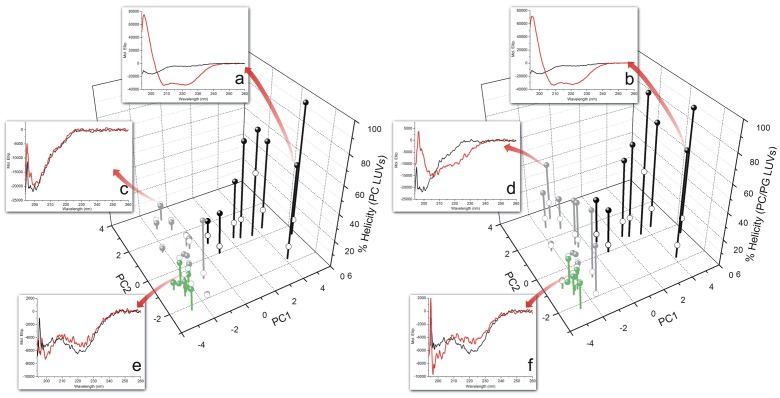
The helicity of peptides in buffer and at 1 mol% DMPC or 2∶1 DMPC:DMPG LUVs are correlated to peptides spatial location in the first two principal components derived from DSC data. Far-UV CD spectra of selected cases for each of the three peptide clusters are shown. Insets (a), (c) and (e) depict the far-UV CD spectra for HSP-4, penetratin and the IAP Q8RW88(70–95), respectively, in buffer (hollow spheres) and titrated with DMPC LUVs (filled spheres). Insets (b), (d) and (f) demonstrate the CD spectra of the same peptides in buffer and added with 2∶1 DMPC:DMPG LUVs. MREs were similar for 0.01 and 0.005 peptide/phospholipids molar ratios, indicating that secondary structure changes reached a plateau under these conditions.

It is interesting to compare the peptide secondary structure changes with the clustering obtained from DSC experiments (Fig. 4). Peptides from Cluster 1 presented random structure in buffer and negligible amounts of secondary structure were detected when LUVs were added, regardless of their composition. Adding DMPC LUVs to peptides of Cluster 2 resulted in no helix formation, except for the peptide Phes. Conversely, the addition of the negatively charged LUVs induced a substantial increase in the helical percentage of all molecules, except for the IAPs B4FGE3(22–37) and A3KLW0(117–136). Peptides from Cluster 3 presented the highest degree of α-helical content when LUVs were added. Moreover, they were almost equally structured in neutral (DMPC) and negatively charged membranes (2∶1 DMPC:DMPG). A high correlation was obtained on the helical percentage of peptides at 1 mol% peptide/DMPC LUVs and the first principal component extracted from DSC data analysis (Fig. S4).

### 4. Effects of Peptides on Model Membranes and their Minimal Inhibitory Concentration (MIC) Towards Pathogenic Bacteria

**Figure 5:**
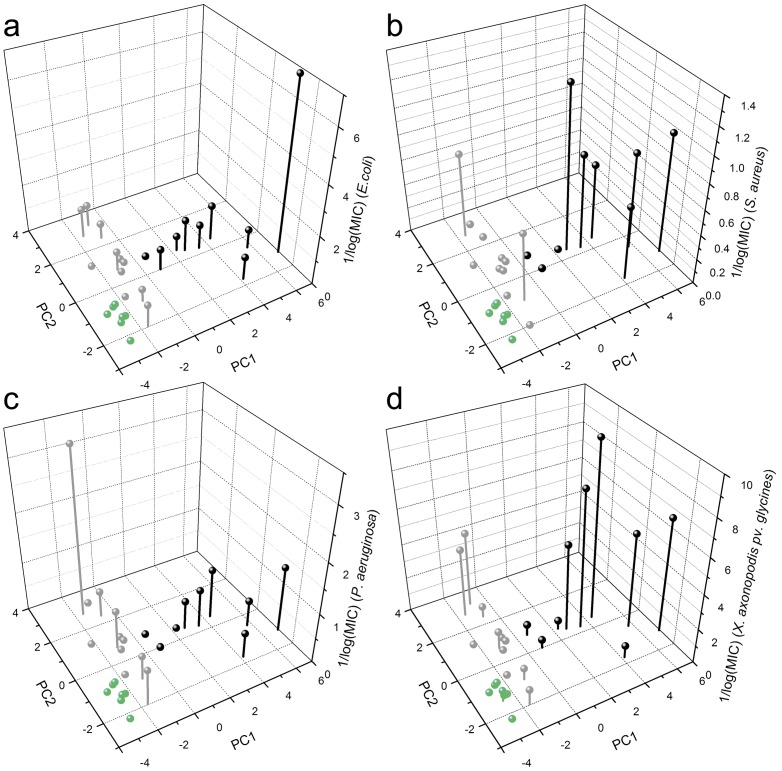
Peptides antimicrobial activities are correlated to their spatial location in the first two principal components derived from DSC data. Antimicrobial activity is represented as the anti-log of the minimum inhibitory concentration (MIC) in micromoles 1/log(MIC(µM)). Peptides MICs can be verified in Table 2. Tested microorganisms were (a) *Escherichia coli*, (b) *Staphylococcus aureus*, (c) *Pseudomonas aeruginosa* and the phytopathogenic bacterium (d) *Xanthomonas axonopodis* pv. *glycines*.

The plot for the first two principal components derived from DSC data as a function of 1/log(MIC(µM)) for *E.coli*, *P. aeruginosa*, *X. axonopodis* pv. *glycines* and *S. aureus* is depicted in Fig. 5. Cluster 1 is populated by molecules that displayed no detectable antimicrobial activity, except for Hyposin HA-6, which had a MIC of 42 µM towards *X. axonopodis pv. glycines* (Table 1). Cluster 2 is more heterogeneous, including penetratin, a well described antimicrobial agent, as well as inactive peptides. Molecules belonging to Cluster 3 were not only the most active towards the three assayed microorganisms, but also had collectively the lowest MIC values (highest 1/log(MIC(µM)), as can be seen on Table S4.

### 5. Inhibition of the Fixation of *P. pachyrhizi* Spores on the Surface of *G. max* Leaves and on Plants Transformed with gb|ABM17058.1|(213–231)

**Figure 6:**
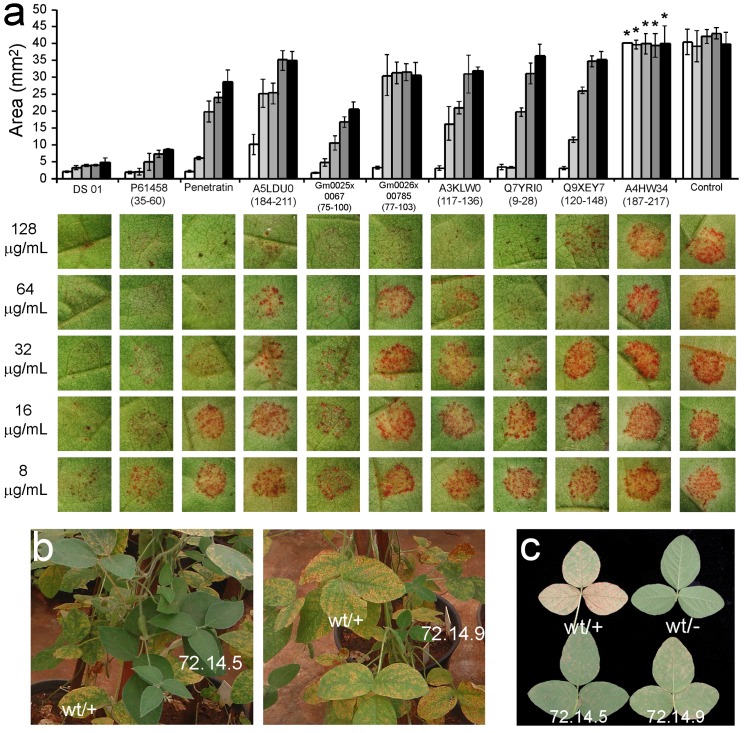
IAPs-induced inhibition of fixation of *Pahakopsora pachyrhizi* spores on soy bean leaves and tolerance of *G. max* transformed with gb|ABM17058.1|(213–231) to the Asian rust in the greenhouse. (a) AMPs and IAPs at 8 to 128 µg/mL were co-incubated with 3.5×10^5^/ml *P. pachyrhizi* spores for seven days on the surface of *G. max* leaves, followed by the estimation of the infected area (represented as a histogram graph). Bars correspond to the sample standard deviation (*n* = 3). All peptides at all tested concentrations resulted in significantly different pustule areas at a 95% confidence interval except for those marked with an asterisk (*). Wild type and *G. max* plants transformed with a vector containing gb|ABM17058.1|(213–231) were grown to the V3 stage and sprayed with a suspension of *P. pachyrhizi* spores (10^6^ spores/mL). (b) Transformed plants (72.14.5 and 72.14.9) were co-cultivated with control plants (wt/+) for 15 days. (c) Control lineages, designated as wt/− and wt/+, correspond to wild-type plants subjected to spraying with water alone or the Asian rust spore suspension, respectively. Intragenic lineages 72.14.5 and 72.14.9 presented a significant reduction in the number of uredia per foliar area (see text).

In order to evaluate if peptides encrypted in *G. max* proteins can confer resistance to the plant’s natural pathogens, two novel IAPs were filtered and synthesized. Gm0025x00667(75–100), primary structure RWRFLRKISSVHMFSVKALDDFRQL, is a fragment of the enzyme flavonoid 3-hydroxylase, while Gm0026x00785(77–103), primary structure HKMDLHWYLRTLEEVVIRALQRFQFR, is derived from the lipoate-protein ligase B. They both inhibited the *in vitro* growth of *X. axonopodis* pv. *glycines*, the causative agent of the bacterial pustule disease, at 5 and 10 µM, respectively. IAPs were also tested *ex vivo* for the inhibition of the fixation of asian rust spores on *G. max* leaves. In general, IAPs co-incubated with spores of *P. pachyrhizi* on the surface of leaves of *G. max* decreased significantly the area occupied by uredias at micromolar concentrations (Fig. 6a). Gm0025x00667(75–100) reduced the infected area to approximately 50% of the control at 8 µg/mL (3 µM).

To test whether IAPs confer resistance to the Asian rust when expressed by plants, *G. max* was transformed with a vector encoding the IAP gb|ABM17058.1|(213–231) [30], [31]. This molecule was conceived by serendipity prior to the systematic attempts to filter, optimize and validate the IAPs described herein. Transformed soybean plants were cultivated along with control lineages to the V3 stage and sprinkled with a suspension of Asian rust spores (Fig. 6b). Two lineages presented a significant decrease in the number of uredia per cm^2^ of foliar area based on the number of pustules observed on expanded leaves 15 days after inoculation (Fig. 6b). The transformed lineages 72.14.5 and 72.14.9 had a reduced number of uredia per foliar area when compared to control plants, from 45±8 pustules/cm^2^ to 7±3 pustules/cm^2^ and 12±3 pustules/cm^2^, respectively.

## Discussion

The present work was motivated and oriented by the empirical observations that a number of proteins house inside their structures peptides that can, individually, display biological activities other than the ones known for their parent proteins. The results described above demonstrate that a methodology based on the *in silico* filtering of putative antimicrobial peptides encrypted in proteins associated with biophysical techniques that evaluate their interactions with model membranes was successfully applied for the identification of eight novel membrane-active peptides showing different levels of membrane interaction tendencies and microbicidal potencies. Furthermore, preliminary data on a possible biotechnological application of such methodology was presented. *G. max* plants transformed with the IAP gb|ABM17058.1|(213–231), a putative antimicrobial fragment from the enzyme D-myo-inositol 3-phosphate synthase, encoded in its own genome, and also considered a stress-related enzyme in plants, was tolerant to the causative agent of the asian rust, *P. Pachyrhizi* [32]. This result was not far from Shewry and Lucas` concept of plant resistance manipulation [33].

The selection of putative IAPs was performed by using the software Kamal, an exploratory tool designed in-house suitable of performing a high-throughput scan in large protein data banks. The filtering procedure results in a subset of protein fragments with higher physicochemical similarity to natural antimicrobial peptides than a random set of molecules from the same database (Fig. 1 and Table 1). However, due to the significant overlap between physicochemical properties of AMP and non-AMP molecules, Kamal cannot be considered an antimicrobial predictor, and the putative IAPs need to be further validated. At this stage, other tools devoted to the prediction of the antimicrobial potential of molecules might be helpful [34], [35]. Kamal can be improved by the incorporation of novel distinctive physicochemical parameters as they become available. A recent study suggests that there are differential patterns of *in vivo* and *in vitro* aggregation potentials in AMPs with respect to non-AMP molecules [36]. Moreover, a better capability to predict IAPs will result from an iterative process that includes the filtering of novel molecules, the evaluation and classification of their modes of interaction with model membranes, and a re-assessment of the physicochemical properties shared by smaller peptide subsets.

The changes on the main phase transition of DMPC and 2∶1 DMPC:DMPG LUVs induced by each investigated peptide yielded a classification system similar to that proposed by McElhaney and Papahadjopoulos *et al.* for lipid-peptide interactions and their relative locations at lipid bilayers [20], [37], [38]. This allows one to estimate the antimicrobial potential of a given molecule prior to antimicrobial assays. In the currently proposed model, increasing alterations on the main phase transition of membranes are associated with projection on the first principal component, as presented in Fig. 3. It is interesting to notice that peptides that interacted exclusively with 2∶1 DMPC:DMPG LUVs had less pronounced effects on the main phase transition of membranes, consistent with the general interpretation that interactions dominated by electrostatic effects lead to more superficial peptide locations, an observation supported by CD data (Fig. 4) [20]. Under these conditions, a broad and significant antimicrobial activity can be considered a natural consequence of a deep interaction between peptides and neutral phospholipids bilayers, attainable by molecules with a high helical propensity in this specific environment. Peptides from cluster 3 displayed such properties, and therefore concur with the hypothesis that a globally amphiphilic conformation is necessary for the disruption of bacterial membranes [39]. Nevertheless, only three IAPs were clustered along with these molecules, indicating that members of this group have a distinctive balance between physicochemical properties that is uncommon in protein fragments.

There are, however, a number of reports of an alternative group of peptides that present significant antimicrobial activity without an amphiphilic structure [40], [41]. These molecules are highly charged and unstructured, and are thought to disrupt biological membranes by clustering away anionic from zwitterionic lipids, inducing membrane defects that increase permeability [42]. Indeed, these peptides do not interact with neutral membranes and require anionic phospholipids for antimicrobial activity. Peptides such as cateslytin and olygo-acyl-lysines (OAKs) are representative examples [42], [43]. It is believed that the promotion of phase segregation is facilitated by the presence of multiple charges in a particular spacing along the primary structure as well as sufficient hydrophobicity to partition into membranes [39]. We propose that the peptides distributed along the second principal component which present significant antimicrobial activity disrupt membranes by such mechanism. The peptides P61458(35–60), A5LDU0(184–211) and penetratin, for example, have borderline hydrophobicity as well as regularly spaced positively charged amino acid residues.

The expression of antimicrobial peptides in plants is known to confer increased resistance to phytopathogens [44]. Indeed, peptides such as esculentin-1, dermaseptin SI and hCAP18/LL37 were used for the transformation of *Nicotiana tabacum*, *Solanum tuberosum* and *Brassica rapa* and conferred resistance to fungal and bacterial pathogens besides providing the plant with insecticidal properties [44], [45], [46]. Preliminary results indicate that *G.max* expressing the IAP gb|ABM17058.1|(213–231), a fragment of the enzyme D-myo-inositol 3-phosphate synthase, presented increased resistance to the Asian rust providing similar results to those obtained with natural antimicrobial peptides (Fig. 6b). It is plausible that IAPs with improved antimicrobial and antifungal spectra are still left undiscovered in the soybean genome, and that novel generations of intragenic plants that are tolerant to a wide array of phytopathogens may be developed.

The proposed strategy of screening bioactive peptides as fragments inside proteins inspired by the natural peptide release and activation under enzymatic proteolysis found across various metabolic processes appears to be universal but, to our best understanding, restricted to certain classes of proteins. Nevertheless, the additional element we modestly hope to append to nature’s magnificent evolutionary plasticity and energy effectiveness exemplified in these processes is the introduction of the theoretical enzyme-free cleavages concept as a complementary mode of revealing biologically active peptides encrypted in protein sequences to the existing physiological and microorganism-based ones. This next step of “oriented-protein processing” does not need to be restrained by enzymatic specificities or optimum cleavage conditions *in vivo* and/or *in vitro* once it may take advantage of the computational predictions capabilities, nucleic acids and peptide synthesis methodologies currently available. The implications of the present findings lead us to: 1. A wider and exciting scenario for bioprospecting new molecules using the ever-growing genomic and proteomic data banks that could be validated by the appropriated bioassays; 2. Expanding the range of existing biotechnological processes applied to dairy and food processing in general; 3. The use of genomic and physiological information from a given species as a possible inner source of new bioactive peptides may represent an inventive alternative to the transgenic technology, once the genetic material to be used belongs to the host organism and not to exogenous sources.

## Materials and Methods

### 1. Materials

DMPC (Dimyristoylphosphatidylcholine) and DMPG (Dimyristoylphosphatidylglycerol) were purchased from Avanti (Avanti Polar Lipids, AL, USA). Peptides are identified by their publication names, by their UniProtKB or genebank (gb|) accession number followed by the indication of the first and last aminoacid residues in brackets. Penetratin, B4FGE3(22–37), A3KLW0(117–136), Q7YRI0(9–28) were purchased from ImmunoKontact (AMS Biotechnology, UK); gb|ACU24018.1|(73–101), gb|AAD22970.1|(120–148) and Q9XEY7(120–148) were purchased from JPT Peptide Technologies GmbH, Germany. The IAPs Q8KG25(327–351), P94692(929–955), P61458(35–60), B0CZJ3(104–130), A4HW34(187–217), Q8RW88(70–95), Q6TV81(25–52), O43312(33–62), A5LDU0(184–211), P83637(1–12) as well as DS 01 (P83637), DS 01(1–12), Nattererin-1 (P86913), Syphaxin (P85279), Phes (HQ012497) HSP-4 (JF916646), Pseudin B (P86915), PS-2 (P84567), Magainin-2 amide (P11006), Hyposin HA-6 (P86921) were synthesized in-house by solid-phase chemistry. Peptides were purified, mass analyzed using an Ultraflex III (Bruker Daltonics, Germany) and quantified by their corresponding molar absorptivities or by the method of Wadell [47].

### 2. Filtering of IAPs

The software Kamal v1.0 *alpha* was written in C++ using the public libraries SQLite, *wxWidgets* and Lua. The application was developed using the IDE code::Blocks and compiled with Mingw3. More details will be given elsewhere. Entries under the label of “frog peptides” were extracted from the Antimicrobial Peptide Database (http://aps.unmc.edu/AP/main.php), resulting in a data set of 487 molecules (Table S1). Net charge, molecular mass, isoelectric point (pI), average hydrophobicity, hydrophobic moment, minimum and maximum on a Kyte-Doolittle hydropathy plot, aggregation potential (Na4vSS) using the AggreScan algorithm and the peptides secondary structure according to the GORIV algorithm were calculated for each molecule [16], [48]. The transmembrane tendency (TM) scale was used for the calculation of the peptideś average hydrophobicity, hydrophobic moment and the Kyte-Doolittle scale was used for the calculation of the minimum and maximum hydrophobicities on a K-D hydropathy plot using a 9 residue window [49], [50]. Isoelectric point was calculated according to pK values extracted from the literature [51]. Average properties were calculated for this data set (Table S1) and used to extract minimum and maximum values for the filtering of IAPs from different databases (Table 1).

### 3. Lipid Vesicles

DMPC and 2∶1 DMPC:DMPG (w/w) were dissolved in chloroform/methanol (3∶1 v/v) at 10 mg/mL, dried as a thin film on a rotary evaporator and left 3 hours under high vacuum. Phospholipids were then dispersed in 20 mM sodium phosphate – NaOH, 150 mM NaCl, pH 7.4 and hand-shaken until the formation of a cloudy solution, which was then passed 19 times through a 100 nm polycarbonate membrane at 30°C for the formation of large unilamelar vesicles (LUVs). Phospholipid concentration was estimated [52].

### 4. Differential Scanning Calorimetry (DSC)

Thermograms were obtained using a VP-DSC (MicroCal Inc., MA, USA) at a temperature range from 10 to 40°C and a scanning rate of 1°C/min. Blank (buffer baseline) thermograms and 0.5 mM DMPC or 2∶1 (w/w) DMPC:DMPG LUVs were acquired as reference. Peptides were added to fresh samples of 0.5 mM LUVs at a concentration of 20 µM (0.04 mol/mol peptide/phospholipids) at room temperature, immediately followed by DSC data acquisition. Each sample was subjected to repeated thermal scans until there were no distinguishable changes in the thermal profile of the main phospholipid phase transition (P’_β_→Lα) between scans. Data were concentration normalized, baseline subtracted (linear connect), and fitted to a non two-state transition with two peaks determined by the user applying the MicroCal Origin™ software. Re-scans for selected cases were acquired using fresh peptide and LUV solutions to check the reproducibility of the data.

### 5. Circular Dichroism

Experiments were conducted on a Jasco-J810 spectropolarimeter (Jasco International Co., Japan). Spectra were acquired at room temperature from 194 to 260 nm as an average of 4 readings using a 0.1 cm path length cell, data pitch of 0.2 nm and a response time of 0.5 s. Data Scans of buffer and 2 mM DMPC and 2∶1 DMPC:DMPG LUVs solutions were acquired and subtracted from each peptide data. Peptides were scanned at a concentration of 20 µM in buffer and then 100 fold excess of DMPC and 2∶1 DMPC:DMPG LUVs were added, resulting in a molar ratio of 0.01 peptide/phospholipids. The spectra were converted to mean residue ellipticity and readings at [θ]_222_ nm and [θ]_208_ were used to estimate α-helix percentages according to two different methodologies [28], [29]. Proximity to equilibrium was verified by scanning peptides added with twice the LUVs concentration, approximately 4 mM.

### 6. Minimum Inhibitory Concentration Assays (MIC)

MICs were determined using the M7-A6 protocol from the Clinical Laboratory Standards Institute (CLSI). *Escherichia coli* ATCC 25922, *Pseudomonas aeruginosa* ATCC 27853, *Staphylococcus aureus* ATCC 29313 were streaked in Mueller-Hinton agar, grown overnight at 37°C, transferred to Mueller-Hinton broth and incubated until readings at 600 nm reached the equivalent of 0.5 in the MacFarland scale. Initial bacterium inoculum of ∼10^5^ colony forming units/mL were transferred, along with serial 2-fold dilutions of each peptide to 96-well plates and incubated for 12 h. Highest peptide concentrations tested was 256 µg/mL. For each peptide concentration, optical density (OD_600_) readings were subtracted from that of the growth medium and divided by the positive control (100% bacterial growth). Inhibitory assays for *X. axonopodis* pv. *glycines* were conducted by the same methodology with longer incubation time (48 h).

### 7. *In situ* Germination Inhibition of *Pahakopsora Pachyrhizi* Spores on *Glycine Max* Leaves’ Surface

*P. pachyrhizi* spores were scraped off infected *G. max* leaves and were frozen at −80°C. Prior to the *in situ* assay, spores were dissolved in Mili-Q^®^ water, incubated at 40°C for 40 min and quantified using a Neubauer chamber. Fifty microliters of spore suspension (3.5×10^5^/ml) were applied to the abaxial surface of leaf disks (16 mm in diameter) detached from middle leaflet of the youngest fully expanded trifoliate leaves from plants at V3 stage (var. BR-16). Spores were then co-incubated with fifty microliters of a peptide solution at concentrations ranging from 8 to 128 µg/mL. Leaves were immediately placed on a Petri dish containing moist filter papers and incubated at 21°C under 12 h-photoperiod and photographed after 7 days. Images were used to measure the infected area using the QUANT v1.0.1 software. Results displayed herein correspond to the average of three separate experiments.

### 8. Plant Transformation and Asian Rust Tolerance Assay

The gene encoding the peptide gb|ABM17058.1|(213–231), primary structure MIKAFKEATKVDKVVVLWTA, was synthesized by Epoch Life Science Inc. (Sugar Land, TX, USA) and cloned into the vector pBluKSPOXDCAHAS replacing the *oxdc* gene [53]. The vector was used to transform soybean plants as previously described [30], [31]. Six intragenic soybean lines were tested for tolerance to Asian rust (*Phakopsora pachyrhizi*). The plants (20 plants per line at V3 stage [54]) were sprayed with a spore suspension (10^6^ spores/mL) and maintained in the greenhouse at room temperature and sprinkling 4 times a day. Leaves were photographed after 15 days and the number of pustules per square centimeter counted.

### 9. Statistical Analyses

Principal Component Analysis was performed using the **princomp** command of the R statistical software (http://www.r-project.org). The data clustering was conducted using the **mclust** command of the MCLUST R Package for normal mixture modeling via EM, model-based clustering, discriminant analysis and density estimation graph algorithm (http://www.stat.washington.edu/mclust/). Three-dimensional plots were created using Origin 7.0 (OriginLab Corp.).

## Supporting Information

Figure S1 **Thermal scans of 2∶1 DMPC:DMPG LUVs enriched with increasing concentrations of DS 01 show the effect of frog skin antimicrobial peptides on the main phase transition of phospholipids.** Non two-state model fitting of the P’_β_→Lα phase transition of a solution of 0.5 mM 2∶1 DMPC:DMPG LUVs enriched with a) pure phospholipids, b) 1 mol% DS 01, c) 2 mol% DS 01 and d) 4 mol% DS 01. Compared with LUVs of the same composition, samples enriched with 4 mol% DS 01 have a sharp component that is shifted to lower temperatures (from 24.3 to 19.3°C) with a lower transition enthalpy and cooperativity (ΔH from 2.2 to 0.3 kcal/mol and ΔH_VH_ from 1000 to 160 kcal/mol). The broad component shifts to higher temperatures (from 23.6 to 25.8°C), has a slightly lower transition enthalpy (ΔH from 2.5 to 1.7 kcal/mol), and becomes even broader (ΔH_VH_ from 340 to 83 kcal/mol). Total enthalpy associated with the main phase transition is decreased to less than half (ΔH from 4.8 to 2.0 kcal/mol). These effects are qualitatively the same as described by the McElhaney group for other antimicrobial peptides [20].
(JPG)

Figure S2 **Peptides induce distinct effects on the thermotropic phase behaviour of DMPC and 2∶1 DMPC:DMPG large unilamellar vesicles.** The thermograms for DMPC added with 4 mol% (a) PS-2 and (b) Q6TV81(25–52) and 2∶1 DMPC:DMPG added with (c) Q8KG25(327–351) and (d) A5LDU0(184–211) are exemplified. Insets contain the fitted parameters for the broad and sharp peak components according to a non-two state transition model with two manually assigned peaks. Shown thermograms were normalized for the lipid sample mass.
(JPG)

Figure S3 **Putative IAPs and antimicrobial peptides are best clustered in three distinct groups.** Optimal data clustering of peptides in the first three principal components obtained from the PCA analysis of data on Table S2 according to the Bayesian Information criterion (BIC) is obtained when three clusters are considered with variable volume, equal shape and variable orientation (VEV). The ellipses superimposed to the classification plot (on the right) correspond to the covariance of the components.
(JPG)

Figure S4 **The relative position of peptides along the first principal component derived from DSC data is linearly correlated to their percentual helicity at 1** **mol% in DMPC LUVs.** The Pearson correlation coefficient indicates a high correlation (r^2^ = 0.86, p<0.0000001) between the relative position of peptides at PC1 and their percentual helicity when titrated with DMPC LUVs. The non-parametric Spearman’s rank correlation coefficient also pointed to a high degree of correlation between both quantities (ρ = 0.72, p = 0.000018).
(JPG)

Table S1 **Physicochemical properties of a sample of frog AMPs obtained from the Antimicrobial Peptide Database** **(** http://aps.unmc.edu/AP/main.php **)** [**15**] **compared to a sample of putative IAPs filtered by Kamal from** ***Glycine max*** **proteins (derived from the Joint Genome Institute - Glyma0.1c.pep.fa.gz)** [**14**] **as well as randomly selected protein fragments from the same** ***G. max*** **database.**
(XLSX)

Table S2 **Numerical data from the fitting of the main phase transition of DMPC and 2∶1 DMPC:DMPG LUVs added with peptides at 4 mol% to a non-two state transition with two components (sharp and broad) and component loadings of the principal component analysis applied to the data.**
(XLSX)

Table S3 **Peptides Mean Residue Ellipcity (MRE) in buffer and titrated with LUVs and their percentual of a perfect helical segment according to the methodology of Chen** ***et al.*** **1974** [**28**] **and Greenfield** ***et al.*** **1969** [**29**] **.**
(XLSX)

Table S4 **Antimicrobial activity expressed as the antilog (MIC(µM)) of peptides according to their clusters and a Kruskal-Wallis statistical test.**
(XLSX)
