# Supplementary figures and images for: Probing Protein Sequences as Sources for Encrypted Antimicrobial Peptides
Source: PLoS One. 2012 Sep 28;7(9):e45848. doi: 10.1371/journal.pone.0045848 (PMC3461044; doi:10.1371/journal.pone.0045848)

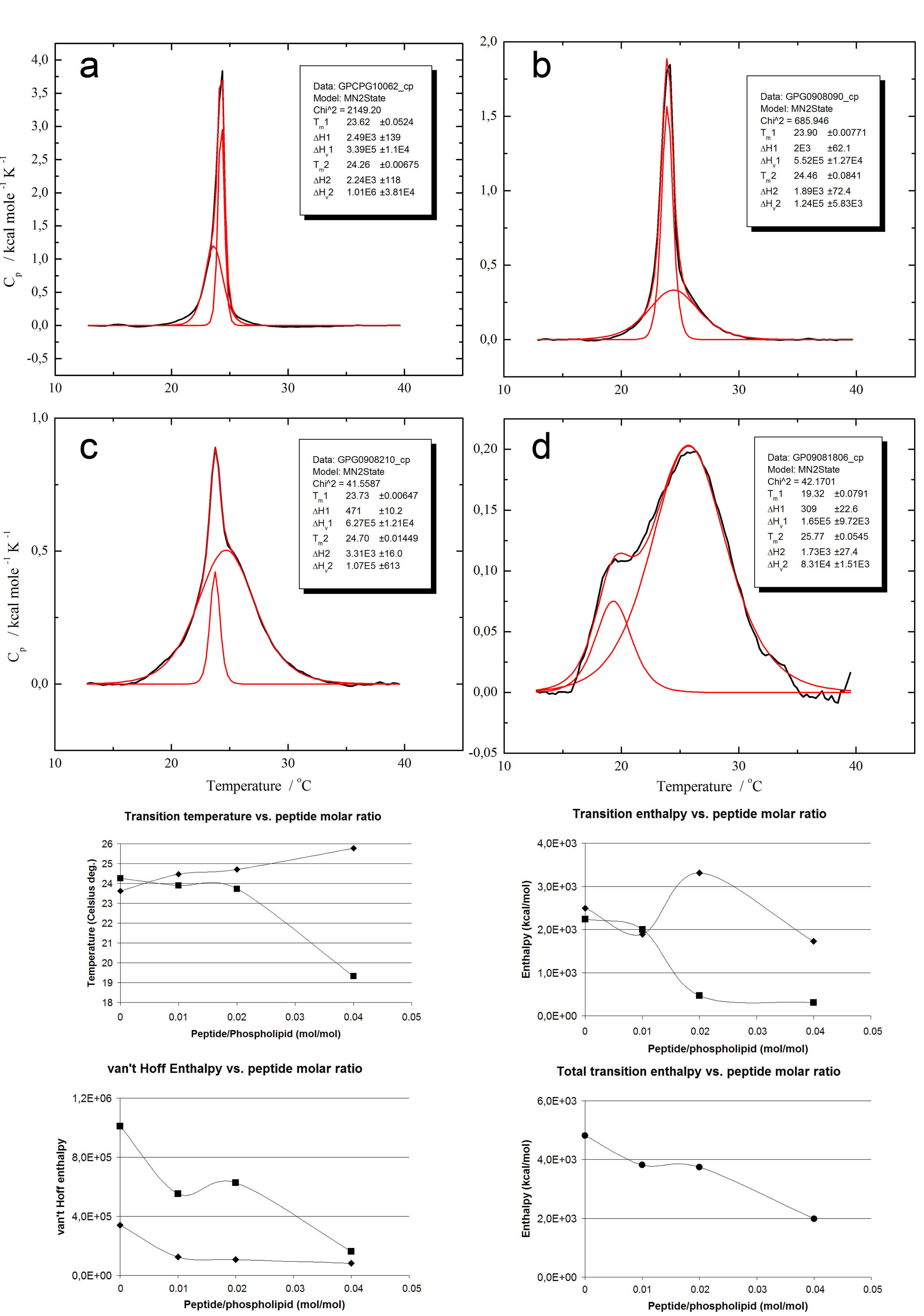

Supplement: Figure S1 — Thermal scans of 2∶1 DMPC:DMPG LUVs enriched with increasing concentrations of DS 01 show the effect of frog skin antimicrobial peptides on the main phase transition of phospholipids. Non two-state model fitting of the P’β→Lα phase transition of a solution of 0.5 mM 2∶1 DMPC:DMPG LUVs enriched with a) pure phospholipids, b) 1 mol% DS 01, c) 2 mol% DS 01 and d) 4 mol% DS 01. Compared with LUVs of the same composition, samples enriched with 4 mol% DS 01 have a sharp component that is shifted to lower temperatures (from 24.3 to 19.3°C) with a lower transition enthalpy and cooperativity (ΔH from 2.2 to 0.3 kcal/mol and ΔHVH from 1000 to 160 kcal/mol). The broad component shifts to higher temperatures (from 23.6 to 25.8°C), has a slightly lower transition enthalpy (ΔH from 2.5 to 1.7 kcal/mol), and becomes even broader (ΔHVH from 340 to 83 kcal/mol). Total enthalpy associated with the main phase transition is decreased to less than half (ΔH from 4.8 to 2.0 kcal/mol). These effects are qualitatively the same as described by the McElhaney group for other antimicrobial peptides [20]. (JPG) [file pone.0045848.s001.jpg]

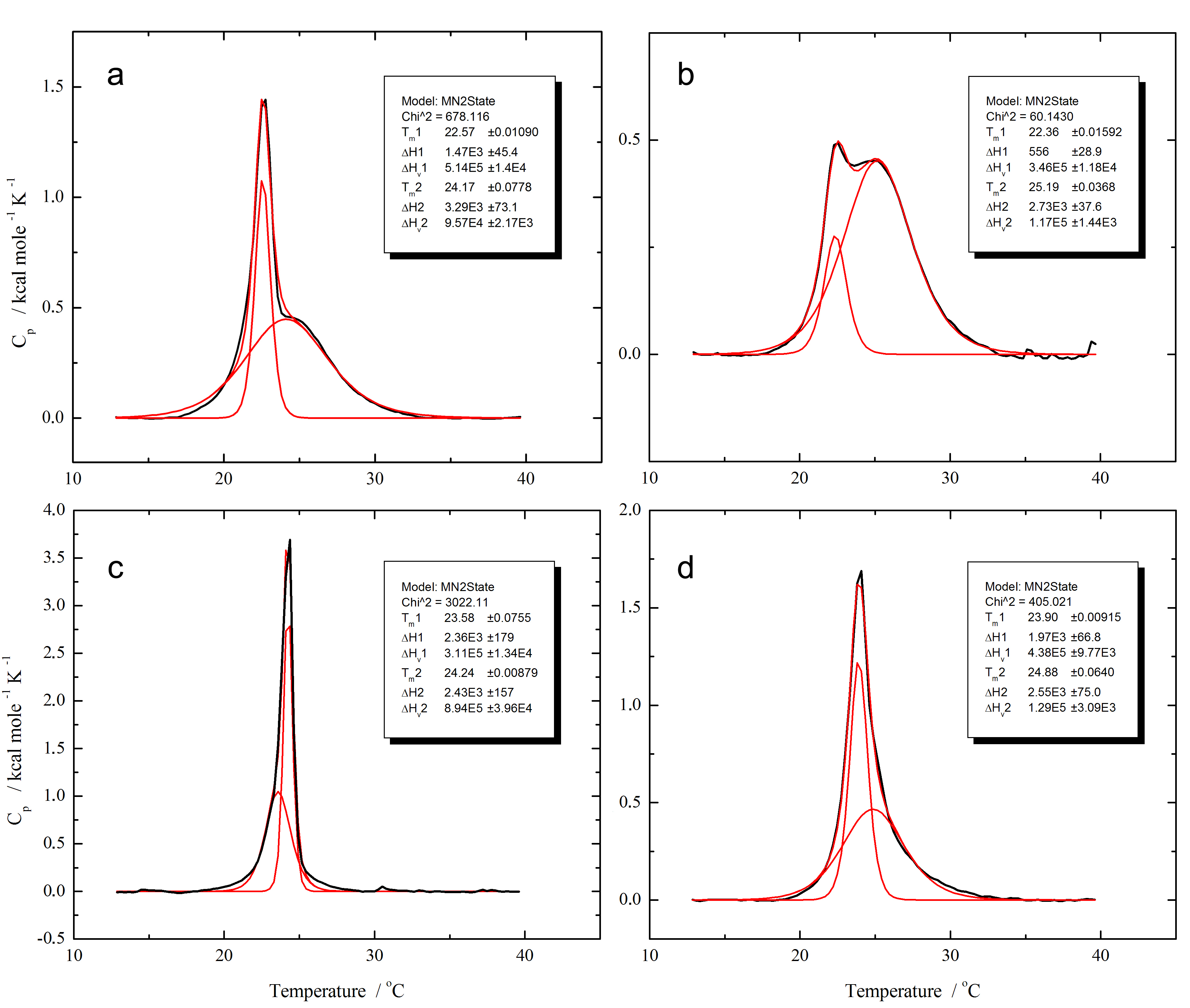

Supplement: Figure S2 — Peptides induce distinct effects on the thermotropic phase behaviour of DMPC and 2∶1 DMPC:DMPG large unilamellar vesicles. The thermograms for DMPC added with 4 mol% (a) PS-2 and (b) Q6TV81(25–52) and 2∶1 DMPC:DMPG added with (c) Q8KG25(327–351) and (d) A5LDU0(184–211) are exemplified. Insets contain the fitted parameters for the broad and sharp peak components according to a non-two state transition model with two manually assigned peaks. Shown thermograms were normalized for the lipid sample mass. (JPG) [file pone.0045848.s002.jpg]

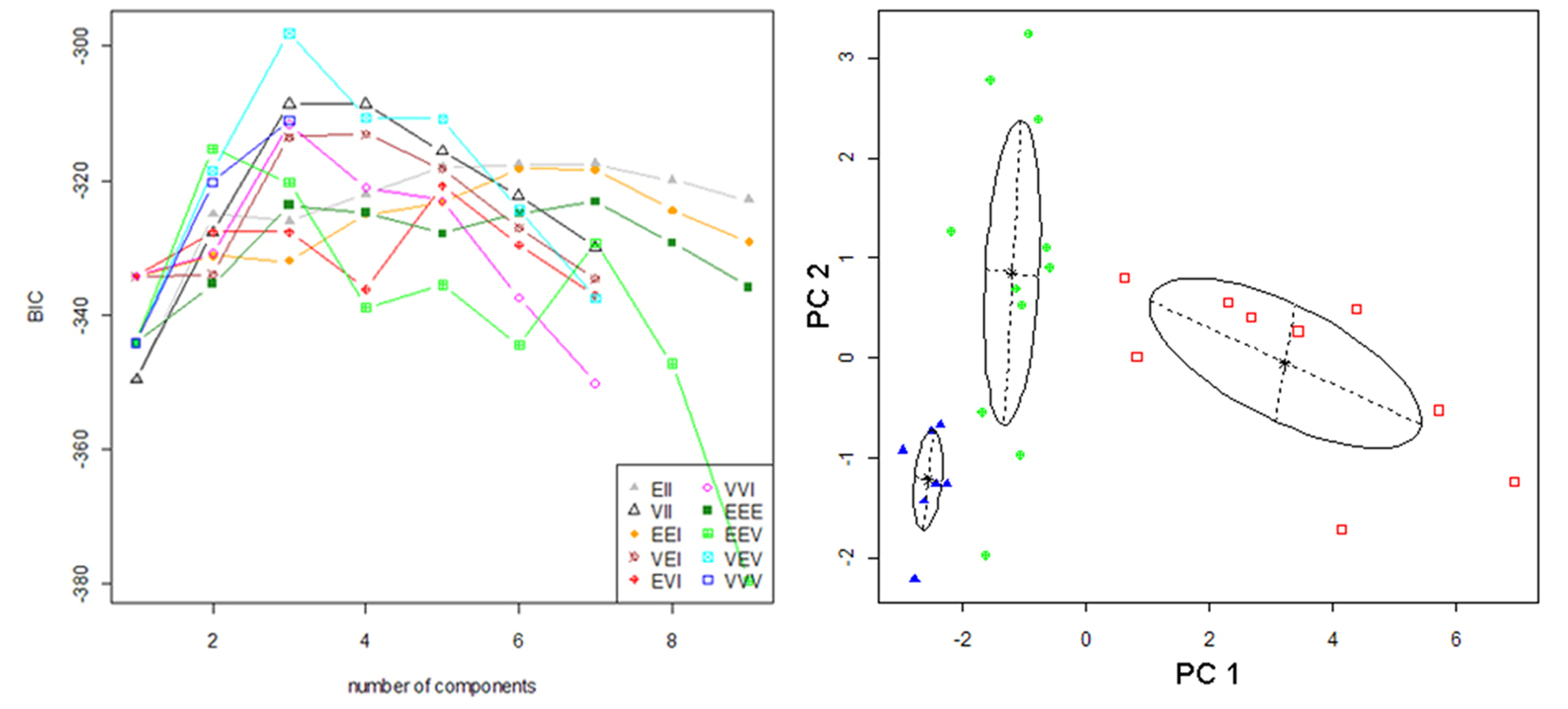

Supplement: Figure S3 — Putative IAPs and antimicrobial peptides are best clustered in three distinct groups. Optimal data clustering of peptides in the first three principal components obtained from the PCA analysis of data on Table S2 according to the Bayesian Information criterion (BIC) is obtained when three clusters are considered with variable volume, equal shape and variable orientation (VEV). The ellipses superimposed to the classification plot (on the right) correspond to the covariance of the components. (JPG) [file pone.0045848.s003.jpg]

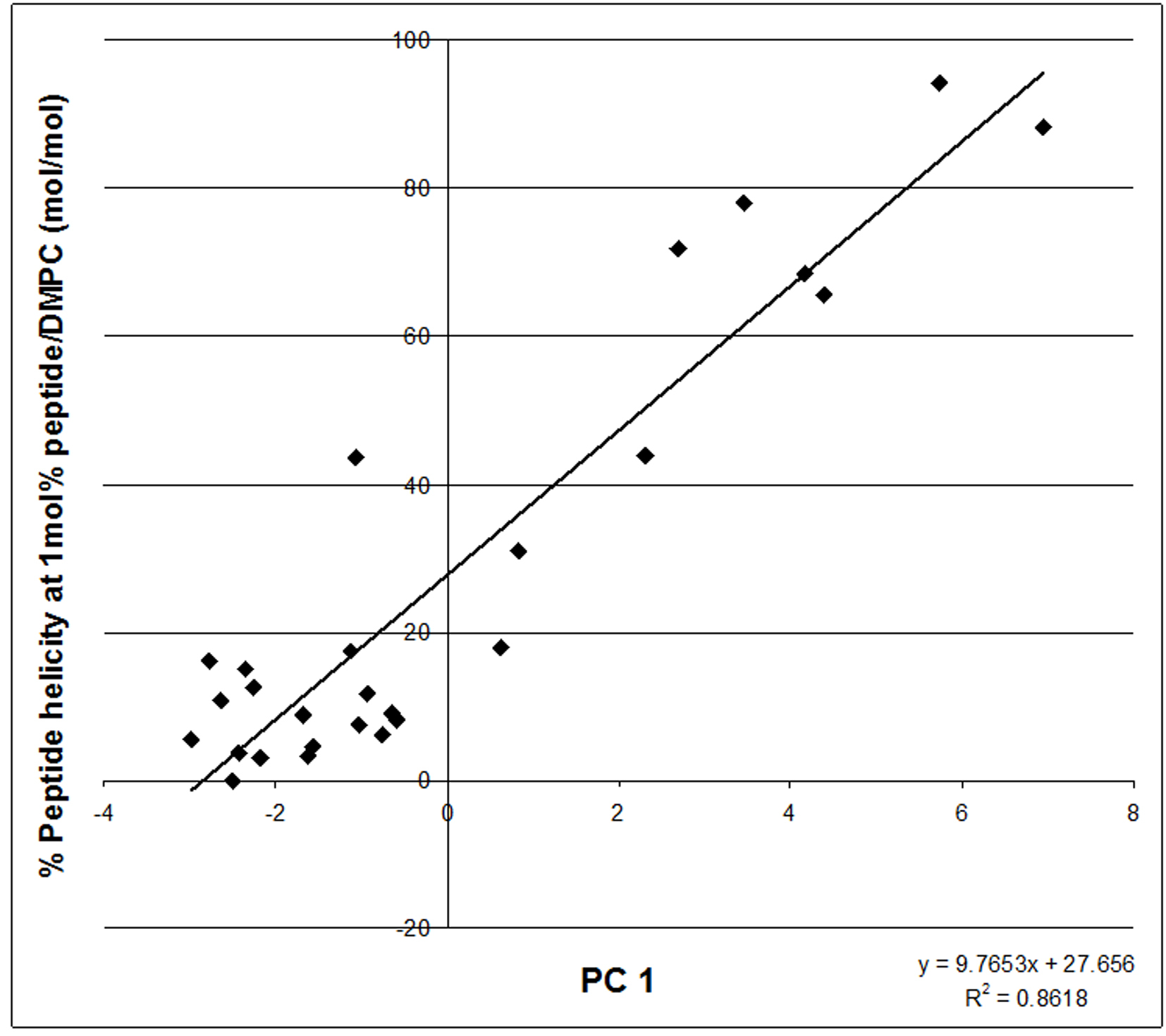

Supplement: Figure S4 — The relative position of peptides along the first principal component derived from DSC data is linearly correlated to their percentual helicity at 1 mol% in DMPC LUVs. The Pearson correlation coefficient indicates a high correlation (r2 = 0.86, p<0.0000001) between the relative position of peptides at PC1 and their percentual helicity when titrated with DMPC LUVs. The non-parametric Spearman’s rank correlation coefficient also pointed to a high degree of correlation between both quantities (ρ = 0.72, p = 0.000018). (JPG) [file pone.0045848.s004.jpg]
